# Early Diagnosis on Oral and Potentially Oral Malignant Lesions: A Systematic Review on the VELscope^®^ Fluorescence Method

**DOI:** 10.3390/dj7030093

**Published:** 2019-09-04

**Authors:** Marco Cicciù, Gabriele Cervino, Luca Fiorillo, Cesare D’Amico, Giacomo Oteri, Giuseppe Troiano, Khrystyna Zhurakivska, Lorenzo Lo Muzio, Alan Scott Herford, Salvatore Crimi, Alberto Bianchi, Dario Di Stasio, Rosario Rullo, Gregorio Laino, Luigi Laino

**Affiliations:** 1 Department of Biomedical and Dental Sciences, Morphological and Functional Images, University of Messina, Policlinico G. Martino, 98100 Messina, Italy; 2 Multidisciplinary Department of Medical-Surgical and Dental Specialties, Second University of Naples, 34109 Naples, Italy; 3 Department of Clinical and Experimental Medicine, University of Foggia, 71122 Foggia, Italy; 4 Department of Maxillofacial Surgery, Loma Linda University, Loma Linda, CA 92354, USA; 5 Department of General Surgery and Medical-Surgery Specialities, University of Catania, 95100 Catania CT, Italy

**Keywords:** oral cancer, oral neoplasms, precancerous conditions, optical imaging, oral surgery, quality of life, diagnostic techniques and procedures

## Abstract

The fluorescence method is an innovative technique used by pathologists for examining body mucosa, and for the abnormalities tissue screening, potentially leading to the earlier discovery of pre-cancer, cancer or other disease processes. The early detection is one of the best mechanisms for enabling treatment success, increasing survival rates and maintaining a high quality of life. The purpose of this review is to evaluate the clinical efficiency of this diagnostic tool applied to the oral cavity (VELscope^®^). A literature systematic review has been performed. The initial research provided 53 results after applying the inclusion and exclusion criteria, and after a manual screening of the abstracts by the authors, only 25 results were eligible for review. The results and data contained in all the researches, no older than 10 years, were manually evaluated, and provided useful information on this diagnostic method. The VELscope^®^ mean value about sensitivity and specificity resulted of 70.19% and 65.95%, respectively, by results analysis, but despite this some studies disagree about its clinical effectiveness, and this diagnostic method is still much debated in scientific and clinical medical literature. Surely being able to have efficient and effective tools from this point of view could help the clinician in the diagnosis, and also make timelier the pharmacological or surgical therapy, improving the quality of life of the patient, and in some cases guaranteeing a longer survival term.

## 1. Introduction

### 1.1. Background

Oral cancer is the eighth most common form of cancer in the world. It represents more than 90% of all malignant neoplasms of the mouth. It affects more often from fifty years of age, and males more often than women, in a ratio of about 2:1. The main risk factors are smoking and alcohol abuse. 

It is highly invasive and debilitating, and represents 3% of all malignant neoplasms in men, and 2% in women. In the last 30 years for women, and today also for young people, this is an increasing phenomenon. Other risk factors are represented by: Smoking and alcohol abuse, bad oral hygiene, poorly designed dental prostheses and the papilloma virus [1,2]. Information on this disease is unfortunately few, and prevention has not yet reached satisfactory levels. Only in Italy every year 8000 people are affected by oral cancer, and more than half of them die in the following five years. A carcinoma intercepted and treated in its initial phase allows for a complete cure and five-year survival in 85% of cases. VELscope^®^ (Visually Enhanced Lesion scope) (LED Apteryx, Akron, OH, USA; Mectron S.p.a., GE, Italy) is a medical tool available in the practitioners’ daily practice, and thus useful for early diagnosis (Appendix A) [3].

It emits a blue light that illuminates the epithelium, and inside it contains a digital camera capable of photographing tissues. Healthy epithelial tissues will appear to be apple green in color, while those that are only slightly damaged will be dark, due to the total loss of fluorescence. This type of visit is painless, and it does not require drugs during the about 10 min it takes. It is divided into two parts: A manual examination carried out by palpating the tissues of the oral cavity, and a visual investigation by illuminating the same fabrics using the light emitted by the VELscope^®^. VELscope^®^ is also recognized by the World Health Organization (WHO) as an efficient tool for the prevention of oral cancer. The most frequent histological type is squamous or spino-cellular cell carcinoma. In the early stages this type of visit is painless, and it does not require drugs during the approximate 10 min duration. The appearance of this squamous or spino-cellular cell carcinoma is of a small superficial red or white lesion (erythroplachia or leukoplakia), or of a non-spontaneous healing ulceration. Sometimes it could take on the appearance of a nodular infiltrative lesion, in the form of a more or less regular thickening detected on the edges, which tends to superficially ulcerate, but also an exophytic or papillary growth form is not infrequent. In some cases, it may present more than one of these aspects simultaneously. The prevailing tendency is for local invasive growth, with rapid lymph node involvement, but a low frequency of distant metastases. The most frequent symptoms are the sensation of increased consistency of the affected mucosa, then the pain, initially in the form of mild but increasing burning, which over time can be accompanied by swelling of the lymph nodes, difficulty in swallowing and difficulty in speech. The final diagnostic test is the biopsy. The therapy for this type of cancer involves the use of surgery or radiotherapy, depending upon the site, while in the more advanced lesions the two techniques are used in combination, sometimes with supportive chemotherapy. The prognosis is usually good, but it greatly depends on the extent of the pathology at the time of diagnosis, therefore quick diagnosis and management are essential. The presence of a large number of benign pathologies capable of confusing with the neoplasm in its early stages, when it is poorly symptomatic, is one of the main causes of the oral cancer late diagnosis. To overcome this problem, various diagnostic techniques have been proposed as screening tests. Among the most promising are: Exfoliative cytology (cytobrush) in its various versions [4,5], vital dyes, the use of particular light emission sources capable of visualizing areas of mucosa with anomalous characteristics through the reactions of chemoluminescence or autofluorescence [3] and, as a recent development, salivary tests and molecular markers [2,6,7,8,9,10,11,12,13,14]. Despite the continuous improvements made over time in order to eliminate the problems of sensitivity and specificity of the various methods, at present there are no certified and predictable routine methods or large-scale screening programs [15,16,17,18].

### 1.2. Objectives

The objective of this systematic review is to evaluate all of the articles present in the recent literature (10 years) concerning the use of the VELscope^®^, therefore assessing its clinical efficiency and evaluating parameters, as sensitivity and specificity. Being a non-invasive and quick-to-use instrument, it could represent a diagnostic method of primary importance for the prevention of the oral cavity.

## 2. Results

### 2.1. Study Selection

The results initially evaluated in this work were 53, and following the application of filters in our scientific database, the number of results was reduced. The studies taken into consideration in this review are not older than 10 years, in order not to compromise the results, which could however be vitiated by a problem related to the VELscope^®^ technology. The published research of the last ten years is only 46, and there are 39 works on humans. In order for the manuscripts to be read and analyzed by the reviewers, it was necessary to include only those available in English and in Full text, which were 32. Following a screening of these thirty-two results, four independent reviewers selected a number of 25 manuscripts.

### 2.2. Study Characteristics

In each paper evaluated, the authors analyzed the results independently. The search for keywords, and the inclusion and exclusion criteria applied to all of these results. The documents all contain information regarding the use of a VELscope^®^, with respect to the use of this diagnostic method for cancerous and precancerous lesions of the oral mucosa.

### 2.3. Risk of Bias within Studies

It is not possible to define a risk of bias between the articles, as not all of them contain sufficient information to do this. The risk of bias within the articles was carried out only for researches containing information therefore.

### 2.4. Results of Individual Studies

The results revealed useful information in the use of VELscope^®^ (Table 1), and thus it is possible to evaluate a synthesis table of the results in the subsequent subsection (Table 2 and Table 3).

### 2.5. Synthesis of Results

### 2.6. Risk of Bias Across Studies

The risk of bias between the articles in this review is difficult to define, as the papers considered do not all contain sufficient information to derive a result. The criteria for assessing the risk of bias cannot always be applied, and during the evaluation of the articles many fields remain empty and do not allow an evaluation. The risk of bias between articles is unclear.

### 2.7. Additional Analysis

**Figure 1:**
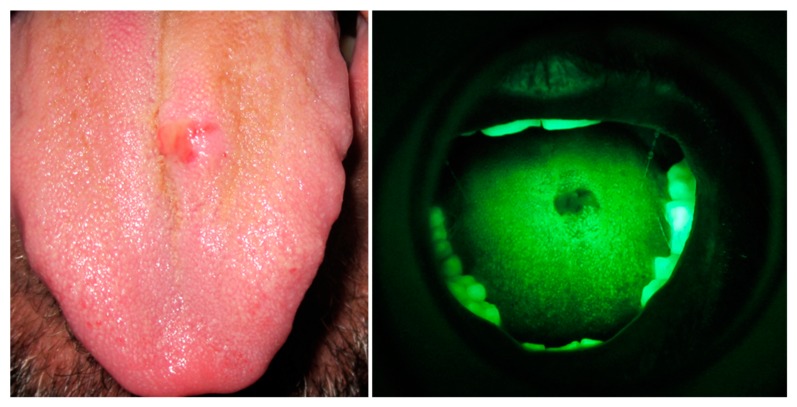
VELscope^®^ results before a tongue biopsy. COE vs. VELscope^®^. For gentle concession of Prof. L. Laino.

**Figure 2:**
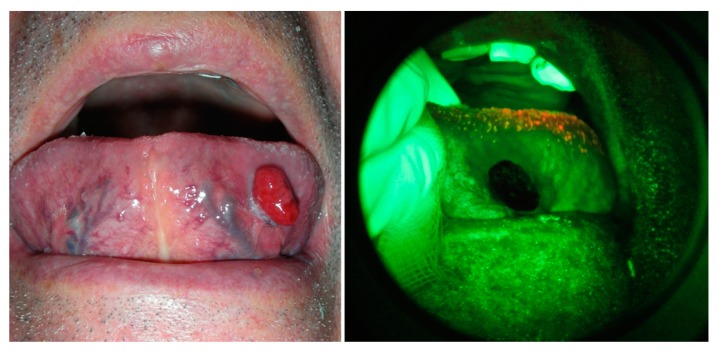
VELscope^®^ results before a tongue biopsy. COE vs. VELscope^®^. For gentle concession of Prof. L. Laino.

In support of the review the authors bring clinical evidence of the use of VELscope^®^; some images are presented by the authors, and are described in the respective captions (Figure 1 and Figure 2). VELscope (r) is an easy-to-use portable instrument, the latter is useful in implementing the specialized dental examination for the search for precancerous or cancerous lesions. Taking advantage of the autofluorescence of the tissues, it is possible that these react to the light emitted in a different way (400–460 nm). The tissue illuminated by an intense blue light expresses a pale green autofluorescence, on the other hand, the pathological tissue on the other hand does not produce light and appears dark, surrounded by healthy tissue.

## 3. Discussion

### 3.1. Summary of Evidence

The use of new diagnostic methods, especially noninvasive ones, can be an incredible advantage in the pathological, clinical field. Furthermore, for these patients, time is of fundamental importance in reducing mortality. The lesions of the oral cavity as already analyzed in chapter 1 represent an important percentage of cancerous lesions. For this reason, the possibility of having a reliable, fast and precise diagnostic tool can be an advantage for the clinician, who daily relates to numerous patients. The studies taken into consideration in this work provide important information to discuss, and fully understand, the advantages and disadvantages of this method, and what aspects could be improved. A recent review regarding the use of VELscope^®^ was carried out by Lingen et al. [44] in 2017. The article by Lingen et al. mainly evaluates the diagnosis of OC. Furthermore, this recent article is a revision of revisions, so a meta-analysis of the results is carried out. This revision also evaluates all of the RCTs and the CTs, offering a broader view of the field of use and the reliability of the VELscope^®^. Is possible to compare VELscope^®^ sensitivity and specificity with other diagnostic methods: Cytologic testing has a 95% specificity and 95% sensitivity range, according to Lingen et al. [44]. The aim of the Farah et al. [19] study was to evaluate molecular differences between autofluorescence and white light-defined margins on oral biopsy. An histology and a molecular biomarkers analysis were performed on biopsy samples [6]. The use of the autofluorescence technique provides a better guide for surgeons. According to Canjau et al. [20] VELscope^®^ or visual fluorescence evaluations cannot replace histopathology for diagnosis. But they could be a simple and a low-cost margin determination method, which method could add the sensitivity of the conventional oral examination. According to Amirchagmachi et al. the fluorescence examination could be used as adjunctive device in specialized centers, but it is not capable to distinguish benign and malignant lesions. According to Yamamoto et al. [22] the VELscope^®^ method could be an auxiliary method for dysplasia diagnosis on oral mucosa and the tongue. Huang et al. [23] evaluated differences in digital camera photos of VELscope^®^ or with light oral cancer, precancerous or normal mucosa. VELscope^®^ could be used, according to the authors, for a diagnosis of oral cancer or precancerous lesions. Ganga et al. [24] evaluated differences between Conventional Oral Examination (COE) and Autofluorescence examination using VELscope^®^. VELscope^®^ results were compared with histopathological results. According to the authors, VELscope^®^ alone could not provide a diagnosis instrument, giving a high number of false positives. It could be used to alleviate patient anxiety on mucosal lesions. 

According to Cicciù et al. [25], the autofluorescence can be used as a helpful method useful to find oral precursor malignant lesions and the correct location for taking biopsies within the altered mucosa. Burian et al. [26] said that VELscope^®^ could help clinician observations in identifying tumor margins on oral mucosa, but it lacks the ability to evaluate any risk of oral lesion. Scheer et al. [27] evaluated the fluorescence properties of oral mucosa lesions. The autofluorescence examination does not provide any additional information to conventional, observational examination. According to Ohnishi et al. [28] the VELscope^®^ diagnosis methods have a high sensitivity and specificity, and could be a device for cost-effective screening or margin detection for biopsy. Nagi et al. [29] evaluated the use of autofluorescence and chemoluminescence for earlier detection of oral squamous cell carcinoma and oral potentially malignant disorders. Both devices are simple and not invasive, but authors do not provide information about its efficacy as to being an early diagnostic tool. Kordbacheh et al. [30] tried to find correlated molecular mechanisms to tissue fluorescence for better clarifying and reduce false positive and false negative VELscope^®^ results. Each examinated lesion types had a specific set of expressed genes; these genes could involve different inflammatory reactions, angiogenesis processes and cell cycle regulations. Inflammatory reaction may be correlated to fluorescence. Rashid et al. [31] evaluated differences between chemoluminescence methods and auto-luminescence ones (VELscope^®^ and MicroLux/DL). 24 articles have been evaluated, according to this systematic review, and some studies illustrated the good sensitivity of this device, but it could provide a high number of false positives. According to Jane-Salas et al. [32], there are no clinical benefits throughout the use of VELscope^®^ on oral lesions diagnosis. Elvers et al. [33] said that VELscope^®^ could diagnose lesions on mucosal inflammation loci, but this method could help clinicians to extend biopsy margins beyond visible margins. Hanken et al. [34] said that VELscope^®^ could be a non-invasive and simple test to diagnose OPMD. According to Rana et al. [35] VELscope^®^ offers a new diagnostic device for OPMD diagnosis compared to white light or conventional examination. McNamara et al. [36] evaluated fluorescent examination compared to conventional oral examination, and they concluded that conventional oral examination is more valid than fluorescent techniques. According to Farah et al. [37] VELscope^®^ helped to find uncovered oral lesions and enhance the visibility of the conventional examination technique. But VELscope^®^ could not provide a definitive diagnosis. According to Scheer et al. [38] this device could help clinicians during the screening phase, but it could not discriminate between benign or malignant lesions. According to a Matsumoto study [39], VELscope^®^ could provide a useful tool for clinicians on the early detection of OPMD and Oral cancer. Lopez-Jornet et al. [40] conducted a literature review, and concluded that VELscope^®^ could not diagnose oral cancer alone, but it could be an adjunct tool. According to Fricain [41] VELscope^®^ needs histological examination to make a diagnosis. Awan et al. [42] evaluated the use of VELscope^®^ for OPMD diagnosis, where they evaluated the use of VELscope^®^ compared to COE and biopsy. According Awan et al. [42] VELscope^®^ can be used by clinicians to evaluate and discover oral mucosal disorders, but it cannot discriminate between lesions’ risk. Mehrotra et al. [43] in 2010 evaluated differences between chemoluminescence and autofluorescence on precancerous or cancerous lesions’ detection. VELscope^®^ could provide false positives and false negatives. The therapeutic choice for oral cancer depends mainly on the staging of the tumor at the time of diagnosis. In the early stages, with limited injury, the therapy of choice is surgical, with low invasiveness. It is always necessary to consider in these cases the presence of noble anatomical structures [45,46,47], which could be affected by the malignant lesion, or which in any case must be respected during surgery. With local progression and expansion, the choice can vary between surgery and radiotherapy, both considered equally valid, depending upon the locations involved. Accordingly, to the recent discoveries, the combined use of the two techniques becomes indicated, also with the supportive chemotherapy. This can be used both after the primary intervention (adjuvant therapy) and as a preventive one (neo-adjuvant therapy), an approach on whose usefulness there is no agreement. The attempt to replace the surgical approach in less advanced cases with less destructive techniques, in order to preserve delicate organs that are difficult to reconstruct [48], does not currently seem to provide promising results. The results also show that the sensitivity of the VELscope^®^ is similar to that of the physical examination in the case of malignant lesions. 

This therefore indicates that the VELscope^®^ could be used in the event that the diagnosis is doubtful, or if the operator is not an expert. In some cases, other lesions could be associated with the primary lesion, and make surgery more complex, or still require reconstructive maneuvers [15,48,49,50,51,52,53,54,55,56,57,58,59,60,61]. The pharmacological protocols to be performed must take into account subsequent therapies or radiotherapy [62,63,64]. In addition, surgery must be aimed at minimizing the risk of dehiscence of the wound or superinfection of the latter [65,66]. In the case of patients suffering from systemic diseases [67,68,69], everything becomes even complicated, which is why early diagnosis is certainly the best way to combat these diseases.

### 3.2. Limitations

The limitations of this study can be represented by the fact that results are works in English and in Full text, so that this could lead to the exclusion of important results. Furthermore, the diagnostic method analyzed by us (VELscope^®^) is still covered by a patent, and some of the analyzed documents could present a defect caused by a conflict of interest or receipt of funds. Furthermore, it is necessary to specify that during the collection of the results by their articles, it was not always possible to pair the results, and so perform an univocal statistic.

## 4. Materials and Methods

### 4.1. Protocol and Registration

**Figure 3:**
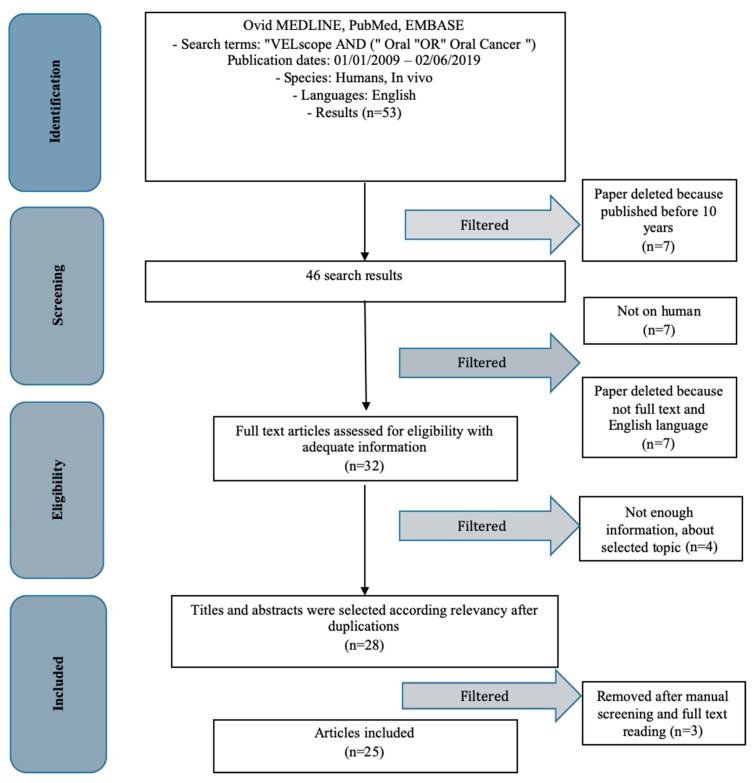
PRISMA flow chart.

The protocol followed is that for the PRISMA reviews (Preferred Reporting Items for Systematic Reviews and Meta-Analyzes), and the checklist, including the flow chart, are those provided by PRISMA (Figure 3). PRISMA aims to help authors improve their reporting of systematic reviews and meta-analyses. Our systematic review was recorded on the PROSPERO website (International prospective register of systematic reviews), which guarantees reliability in the method used for the purposes of the review. PROSPERO includes protocols for health and social care, welfare, public health, education, crime, justice and international development, where there is a health-related outcome. The number and date of the registration protocol are: 137,822 on 04/06/2019.

### 4.2. Eligibility Criteria

The inclusion and exclusion criteria applied have been divided into this subsection and are as follows:

Inclusion criteria:

- VELscope^®^ diagnostic tool study
- VELscope^®^ Randomized Controlled Trial (RCT) or Clinical Trial (CT)
- Human studies

Exclusion criteria:

- Patients involving systemic or syndromic diseases
- Declared a conflict of interest
- Not enough information about VELscope^®^
- Animal studies
- Older than 10 years studies
- Not accessible title or abstract

### 4.3. Information Sources

The sources of information of this study are represented by the largest scientific databases, the search for keywords, was conducted on PUBMED, EMBASE, SCOPUS and on the search engine MDPI (Multidisciplinary Digital Publishing Institute). The filters used are those provided by search engines, to help with manual screening when necessary.

### 4.4. Search

The keywords applied in the scientific databases (see Section 4.3) are the following: “VELscope^®^ AND (“Oral “OR” Oral Cancer”)”.

According to PICO (Population, Intervention, Comparison, Outcome) guidelines the main question of this review is:

Are VELscope^®^ apparati more accurate in diagnosing Oral Lesions compared with Conventional oral examination (COE) for Specificity and Sensitivity?

And as an alternative question:

For Dental Patients, does the use of VELscope^®^ reduce the future risk of premalignant and malignant oral lesions compared to COE?

### 4.5. Study Selection

The filters applied to screen the results have seen the use of the functions:

- Last 10 years
- English language studies
- Humans
- Full text

### 4.6. Data Collection Process

The subsequent selection of the articles saw the application of inclusion and exclusion criteria and the search for key words in the resulting articles (“VELscope^®^” OR “Oral”). Four authors independently (LF, GT, SC and GC), of four different universities, dedicated themselves to the screening of the results and drew their conclusions, and when in disagreement, the results were analyzed by a fifth expert author of a fifth university (ASH). The supervision of the study was conducted throughout the screening and review phase (Figure 3).

### 4.7. Items

The results revealed useful information in the use of VELscope^®^. The parameters evaluated are the following (Table 1):

- Authors (years)—Author name and year of publication
- VELscope^®^ investigation—VELscope^®^ use and compared group
- Oral Pathology—Information about clinical condition and examined sample
- Results—Synthesis of results
- Statistical analysis—Significant or not results
- Sample size—study analyzed sample size

Subsequently authors analyzed the weighted average on sensitivity and sensibility of VELscope^®^ use and the field of use of this tool.

### 4.8. Risk of Bias in Individual Studies

An individual risk of bias evaluation has been performed [70,71,72,73,74,75] (Table 4):

### 4.9. Summary Measures

All of the statistical data concerning the sensitivity of the use of the VELscope^®^ have been summarized. Therefore, a weighted average of the results was conducted, comparing it to the number of patients. The ranges of the values have been specified (Table 2).

### 4.10. Additional Analyses

As an additional analysis we have supported the revision of the instrumental tests obtained with VELscope^®^, so it has been supported as to help readers to understand clinically its importance and the type of results obtained with this diagnostic method. Surely the presence of these images, in addition to making the function of the VELscope^®^ better understood, can act as an input for the application of this diagnostic method in other fields of medicine that are not yet affiliated (Figure 1 and Figure 2).

## 5. Conclusions

In conclusion, from the results, despite these being conflicting, the VELscope^®^ represents an excellent tool to make diagnoses of lesions of the oral mucosa. Unfortunately, this instrument does not have the capacity to discern between a benign lesion, a malignant one, or a simple acute inflammation. Like any instrument, it requires the experience of the clinician or surgeon for good functioning. This diagnostic tool requires a learning curve. 

However, it has proved to be an excellent tool to guide surgery, and has good sensitivity results. The gold standard for the diagnosis of these injuries is always the biopsy, and so the early diagnostic phase should instead be conducted by a clinician, with a conventional intraoral visit, and VELscope^®^ use could represent a useful tool.

## Figures and Tables

**Table 1:** Synthesis of results.

Authors (Year)	VELscope Investigation	Oral Pathology	Results	Statistical Analysis	Sample Size
Farah et al. (2018) [19]	Differences between white light and autofluorescence on oral potentially malignant disorders (OPMD) detected margins.	Oral epithelial dysplasia, oral lichen planus, oral lichenoid dysplasia	Autofluorescence determined margins are safer		11
Canjau et al. (2018) [20]	Conventional oral examination vs. visual fluorescence evaluation with VELscope^®^	Malignant lesions and premalignant lesions	Fluorescence examination cannot replace histopathology, but it may add sensitivity to the conventional examination		18
Amirchagmaghi et al. (2018) [21]	Fluorescence evaluation in patients with oral lesions	Dysplasia and oral carcinoma	This method is not capable to distinguish malignant from benign lesions.		45
Yamamoto et al. (2017) [22]	Detection accuracy of VELscope on epithelial dysplasia of the tongue	Leukoplakia with and without dysplasia	Autofluorescence visualization could be an auxiliary method for diagnosis	Luminance ration on malignant lesion: *p* < 0.0001	79
Huang et al. (2017) [23]	White light vs. VELscope^®^ method	Oral cancer, precancerous lesions	Fluorescence examination could be used to differentiate oral cancer and precancerous lesions from normal mucosa.		140
Ganga et al. (2017) [24]	Conventional Oral examination vs. VELscope method		Fluorescence examination could not provide a definitive diagnosis	76% of specificity and sensitivity	200
Cicciù et al. (2017) [25]	Tissue fluorescence imaging vs. conventional oral examination or biopsy	Oral squamous cell carcinoma	The main criticism of autofluorescence on performing a diagnosis of cancer was the impossibility of discriminating high-risk from low-risk lesions, but it could be a quick and noninvasive method.		1
Burian et al. (2017) [26]	Tissue fluorescence examination VELscope^®^	Oral soft tissue lesions or carcinoma in situ	VELscope^®^ could help clinicians and help to define biopsy margins	Sensitivity of VELscope *p* = 0.007	90
Scheer et al. (2016) [27]	Tissue fluorescence examination VELscope^®^	Oral squamous cell carcinomas	VELscope^®^ reveals no additional information to analysis	Sensitivity and specificity were 33.3% and 88.6%	41
Ohnishi et al. (2016) [28]	Tissue fluorescence examination VELscope^®^	Carcinoma in situ	Fluorescence examination could represent a simple, cost-effective screening.	Sensitivity 95% and specificity 100%	17
Nagi et al. (2016) [29]	Chemoluminescence and tissue autofluorescence in detection of Oral Squamous Cell Carcinoma (OSCC) and OPMD	OSCC and OPMD	Chemoluminescence and autofluorescence are simple and not invasive tests.	Sensitivity range 22% to 100% and specificity 16% to 100%	Review
Kordbacheh et al. (2016) [30]	Molecular pathways associated with fluorescence properties of OPMD	OSCC, Oral epithelial dysplasia (OED), Oral lichen planus (OLP), Oral epithelial hyperplasia (OEH)	Uncovering these molecular mechanisms could provide a reduction in false positive or negative findings with VELscope^®^		42
Rashid et Warnakulasuriya (2015) [31]	Chemoluminescence vs. tissue autofluorescence in detection of OPMD	OPMD	VELscope^®^ may detect erythematous lesions or benign inflammations as a false positive		Review
Jane-Salas et al. (2015) [32]	Conventional oral examination vs. Autofluorescence technique VELscope^®^	Oral lesions	No clinical benefits were obtained using the VELscope^®^ system	Not significant results	60
Elvers et al. (2015) [33]	Photo examination vs. autofluorescence examination VELscope^®^	OPMD	This technique enables clinicians to measure the extent of lesions beyond visible margins		20
Hanken et al. (2013) [34]	Early detection with VELscope^®^	OPMD	VELscope^®^ is a simple noninvasive way to find OPMD	Sensitivity 22% specificity 8.4%	120
Rana et al. (2012) [35]	Autofluorescence examination vs. white light examination	OPMD	VELscope^®^ is a useful new diagnostic device for the detection of oral cancer diseases.	Sensitivity 100% specificity 74%	289
McNamara et al. [36]	COE vs. Fluorescence examination VELscope^®^	OPMD	Biopsy does not confirm VELscope diagnosis	Scalpel biopsy vs. VELscope^®^ *p* = 0.0001	42
Farah et al. (2012) [37]	Tissue autofluorescence VELscope^®^ in oral mucosa lesions detection	OPMD	VELscope^®^ cannot provide a definitive diagnosis alone	Sensitivity 30% Specificity 63%	112
Scheer et al. (2011) [38]	Autofluorescence evaluation VELscope^®^	OPMD, OSCC, squamous intraepithelial neoplasia	VELscope^®^ can assist the clinician during the identification of OPMD, but it does not help in discriminating benign or malignant conditions	Sensitivity 100%, specificity 80%	64
Matsumoto (2011) [39]	Autofluorescence evaluation VELscope^®^	OSCC, moderate and severe epithelial dysplasia lesions, mild dysplasia lesions and lichen planus.	VELscope^®^ could be a valuable tool in an early detection of potentially malignant and malignant lesions in oral mucosa.		74
Lopez-Jornet et al. (2011) [40]	Autofluorescence evaluation VELscope^®^	Oral cancer	This device needed a conventional oral examination too	Sensitivity 98% to 100% Specificity 94% to 100%	Review
Fricain (2011) [41]	Autofluorescence evaluation VELscope^®^ vs. spectroscopy	OPMD	Histological examination remains the gold standard of OPMD diagnosis, VELscope^®^ could only support clinicians	Sensibility 78% to 100% Specificity 75% to 100%	Review
Awan et al. (2011) [42]	Autofluorescence vs. COE and oral biopsy	Oral leukoplakia, oral erythoplakia, oral lichen planus, hyperplastic candidiasis, rest frictional keratosis, oral sub-mucous fibrosis	VELscope^®^ is not able to discriminate high or low risk lesions, but it can diagnose mucosal disorders	Sensibility 84.1% Specificity 15.3%	126
Mehrotra et al. (2010) [43]	Autofluorescence techniques VELscope^®^ vs. chemoluminescence ViziLite	OPMD	VELscope could provide a false negative	Sensitivity 50% Specificity 38.9%	102

**Table 2:** Statistical data results.

1342 Patient’s Data	Sensitivity or Sensibility	Specificity
VELscope^®^ range	22% to 100%	8.4% to 100%
VELscope^®^ weighted average	70.19%	65.95%

**Table 3:** Field of use.

VELscope^®^	Oral leukoplakia, oral erythoplakia, oral lichen planus, hyperplastic candidiasis, rest frictional keratosis, oral sub-mucous fibrosis, oral squamous cell carcinoma, moderate and severe epithelial dysplasia lesions, mild dysplasia lesion, squamous intraepithelial neoplasia, oral carcinoma in situ

**Table 4:** Risk of bias.

Authors (Year)	Risk of Bias			
	Unclear	Low	Moderate	High
Farah et al. (2018) [19]			x	
Canjau et al. (2018) [20]			x	
Amirchagmaghi et al. (2018) [21]			x	
Yamamoto et al. (2017) [22]			x	
Huang et al. (2017) [23]		x		
Ganga et al. (2017) [24]	x			
Cicciù et al. (2017) [25]				x
Burian et al. (2017) [26]			x	
Scheer et al. (2016) [27]			x	
Ohnishi et al. (2016) [28]	x			
Nagi et al. (2016) [29]				x
Kordbacheh et al. (2016) [30]			x	
Rashid et Warnakulasuriya (2015) [31]			x	
Jane-Salas et al. (2015) [32]	x			
Elvers et al. (2015) [33]			x	
Hanken et al. (2013) [34]			x	
Rana et al. (2012) [35]				x
McNamara et al. [36]	x			
Farah et al. (2012) [37]			x	
Scheer et al. (2011) [38]			x	
Matsumoto (2011) [39]			x	
Lopez-Jornet et al. (2011) [40]	x			
Fricain (2011) [41]			x	
Awan et al. (2011) [42]			x	
Mehrotra et al. (2010) [43]	x			

**Figure A1:**
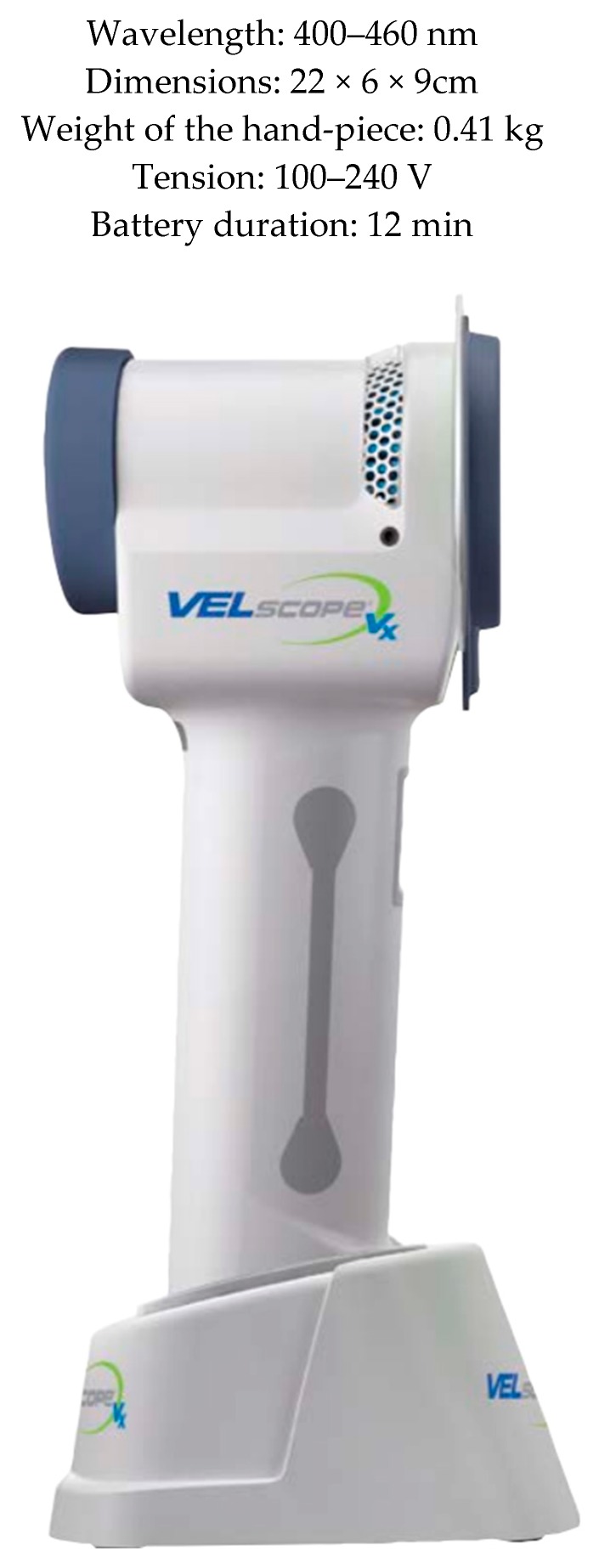
VELscope VX model.

## Appendix A

By radiating the tissues of the oral cavity with a high intensity light, the natural fluorescence of the tissues is stimulated. Light stimulates various molecules in our cells, making them absorb light energy, which is then re-emitted through fluorescence. However, this fluorescence is covered by reflected light, and cannot be seen by the human eye; if white light is filtered, the fluorescence becomes visible instead. Variations in the natural fluorescence of healthy tissues generally reflect biochemical or structural changes that may indicate suspicious lesions [76,77,78,79,80].

The technical characteristics of the VELscope^®^ are the following (Figure A1):
